# Selective transplantation method of leafy vegetable seedlings based on ResNet 18 network

**DOI:** 10.3389/fpls.2022.893357

**Published:** 2022-07-22

**Authors:** Xin Jin, Lumei Tang, Ruoshi Li, Jiangtao Ji, Jing Liu

**Affiliations:** ^1^College of Agricultural Equipment Engineering, Henan University of Science and Technology, Luoyang, China; ^2^Collaborative Innovation Center of Machinery Equipment Advanced Manufacturing of Henan Province, Luoyang, China; ^3^Science & Technology Innovation Center for Completed Set Equipment, Longmen Laboratory, Luoyang, China

**Keywords:** deep learning, transfer learning, seedling screening, seedling characteristics, machinery automation

## Abstract

To solve the problem of low survival rate caused by unscreened transplanting of seedlings. This study proposed a selective transplanting method of leafy vegetable seedlings based on the ResNet 18 network. Lettuce seedlings were selected as the research object, and a total of 3,388 images were obtained in the dataset. The images were randomly divided into the training set, validation set, and test set in the ratio of 6:2:2. The ResNet 18 network was used to perform transfer learning after tuning, identifying, and classifying leafy vegetable seedlings, and then establishing a model to screen leafy vegetable seedlings. The results showed that the optimal detection accuracy of the presence and health of seedlings in the training data set was above 100%, and the model loss remained at around 0.005. Nine hundred seedlings were selected for the validation test, and the screening accuracy rate was 97.44%, the precision rate of healthy seedlings was 97.56%, the recall rate was 97.34%, the precision rate of unhealthy seedlings was 92%, and the recall rate was 92.62%, which was better than the screening model based on the physical characteristics of seedlings. If they were identified as unhealthy seedlings, the manipulator would remove them during the transplanting process and perform the seedling replenishment operation to increase the survival rate of the transplanted seedlings. Moreover, the seedling image is extracted by background removal technology, so the model processing time for a single image is only 0.0129 s. This research will provide technical support for the selective transplantation of leafy vegetable seedlings.

## Introduction

In China, with the accelerating pace of industrialization and urbanization, the production and supply capacity of vegetable agricultural products are facing resource and environmental constraints (Huang et al., 2022). Fully relying on the progress of agricultural science and technology and continuously improving the rate of land output has become the inevitable choice to ensure the safety of China’s vegetable supply. Therefore, there is an urgent need to develop mechanized and automated production of soilless cultivation and promote the transformation and upgrading of the vegetable industry. Due to the protection and link controllability of protected horticulture, the output value of protected horticulture agriculture is 2 ~ 4 times higher than that of open-field agriculture.

This paper mainly studies the greenhouse seedling transplanter. Research on how to improve the transplant efficiency, transplant precision, reduce the seedling damage rate, and ensure the survival rate after transplantation is the key to studying the seedling transplant machinery, which is of great significance to improve the agricultural production of protected horticulture. The quality of the seedlings and the performance of the seedling transplanting robot determine the quality, efficiency, and reliability of the transplanting. However, there are problems in the batch seedling raising, such as missed seeding, rotten seeds, and poor growth of seedling leaves. Normal seedlings account for about 80–95% of the total number of seedlings (Chen, 2000). If the seedlings are transplanted directly without grading screening, the survival rate of the transplanted seedlings cannot be guaranteed.

Machine technology is widely used in assisting human eyes in identifying fruits and vegetables, crop grading, crop diseases, and insect pest identification (Yang et al., 2016; Fan et al., 2020; Fu et al., 2020; Greener et al., 2021). Zou et al. (2021) Machine learning combined with color and shape features was used to identify and screen wormholes on broccoli leaves in the wild environment, and the ratio of wormhole area to broccoli leaf area was calculated. The harm degree of wormholes was evaluated to provide a reference for precise spraying of pesticides. Kurmi and Gangwar (2021) proposed a Leaf Image Localization-Based Algorithm for Different Crops Disease Classification.

Deep learning is a kind of machine learning which has become a popular technology in various fields in recent years, such as image processing, speech recognition, and machine translation (Sharma et al., 2019; Weng et al., 2019; Azimi et al., 2020; Chen et al., 2020; Sao et al., 2020; Zhu and Zheng, 2020). Tu et al. (2021) proposed a non-destructive and highly efficient model for detecting the genuineness of maize variety “Jingke 968” using machine vision combined with deep learning. The VGG16 network was used for transfer learning after fine-tuning to identify and classify the seed images.

Yoosefzadeh-Najafabadi et al. (2021) proposed a Hyperspectral Vegetation Index based on artificial intelligence and an evolutionary optimization algorithm to estimate soybean yield and fresh biomass, which soybean breeders can employ for discriminating superior genotypes in large breeding populations. Li et al. (2021) proposed an automatic detection method for Hydroponic Lettuce Seedlings based on the improved Faster RCNN framework to detect the dead and double-planting status of seedlings during the growth period. The average accuracy of this method for hydroponic lettuce seedlings is 86.2%. Sun et al. (2019) proposed a crop detection method based on the Faster R-CNN model; Broccoli seedling images in different environments were collected to establish data sets; the results showed that the ResNetl01 network was the best feature extraction network. The average detection accuracy was 90.89%, and the average time-consuming was 249 ms.

He (2019) identified tobacco seedlings through machine vision and deep learning technology, and the images of tobacco seedlings on the plug tray were shot from a top view in a closed space. The CNN model algorithm was used to identify the single plant, multiple plants, and cavity trays, and the identification accuracy rate of vigorous seedlings can reach 99.05%. Jin et al. (2021) proposed a threshold optimization method based on a genetic algorithm and a three-dimensional block matching algorithm (BM3D). The leaf area of potted seedlings was measured by machine vision technology, and the growth status and location information of potted seedlings were detected. An intelligent identification framework for healthy vegetable seedlings (SIHVS) was constructed to identify healthy potted seedlings; the recognition accuracy of this method was 94.33%.

**Figure 1:**
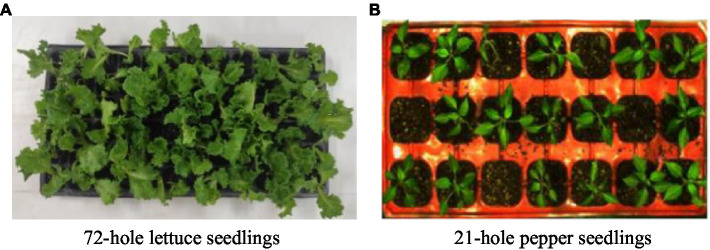
Application object comparison. **(A)** 72-hole lettuce seedlings. **(B)** 21-hole pepper seedlings.

The method proposed by Jin et al. (2021) was used to identify the healthy seedlings of lettuce seedlings, but it was not practical for identifying them. As shown in Figure 1, a comparative image of lettuce seedlings and pepper seedlings is shown. The main reasons for the poor identification of lettuce seedlings are as follows: (1) The dimension of the plug tray is 540 mm × 280 mm. The application object is pepper seedlings cultivated in 21-hole plug trays. The plug tray holes are large, and the segmentation effect is better. However, lettuce seedlings are cultivated in 72-hole plug trays, the size of the plug trays is small, and the leaves of adjacent seedlings are staggered, making it difficult to separate a single seedling. (2) Pepper seedlings have main stems and slender leaves; between adjacent seedlings, leaves are less staggered, which are conducive to segmentation. However, lettuce seedlings have no main stems and belong to leafy crops, and the leaves are widened, which are not conducive to segmentation.

To sum up, due to the large leaves of leafy vegetable seedlings during the transplant period, it is difficult to segment a single seedling for healthy seedling screening by collecting images from a top view, resulting in unhealthy seedlings also being transplanted into the cultivation trough, which reduces the survival rate after transplantation. Therefore, a selective transplantation strategy based on deep learning is proposed to screen healthy seedlings in the process of seedling transplantation, eliminate unhealthy seedlings, and ensure the consistency of seedlings in the cultivation trough, to improve the survival rate after seedling transplantation, which is of great significance to leafy vegetable seedling transplantation.

This paper is divided into four sections: The first section is the Introduction, which introduces the research background and the work done in this paper. Section “Materials and methods”: Taking lettuce seedlings as the test object, the selective transplantation method, image acquisition, seedling extraction, and deep learning algorithms are introduced; A leaf vegetable seedling screening model based on ResNet 18 transfer learning network and a leaf vegetable seedling physical characteristics screening model was proposed. Section “Results and discussion” introduces the training results of the seedling screening model and discusses the advantages and limitations of this study. Section “Conclusion” summarizes the key findings of this study and speculates on the future research direction.

## Materials and methods

### Screening method

**Figure 2:**
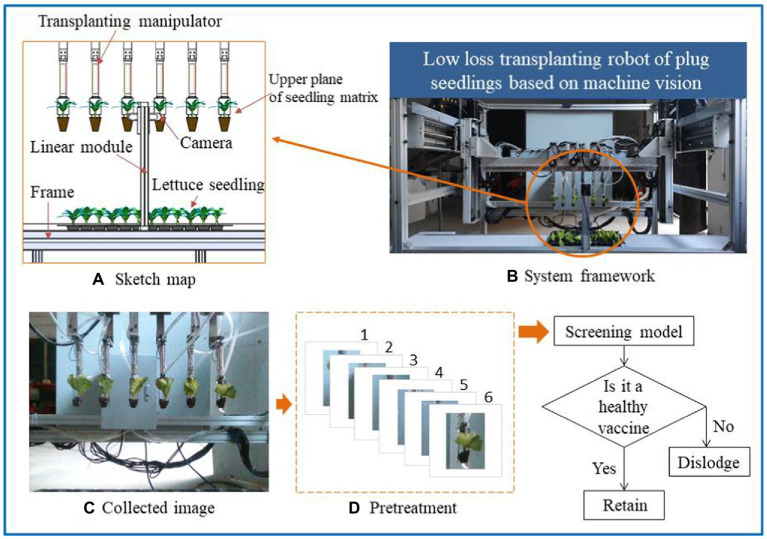
The theoretical approach to selective transplantation of leafy seedlings. **(A)** Sketch map. **(B)** System framework. **(C)** Collected image. **(D)** Pretreatment.

The study is carried out on the low-loss seedling transplanting robot based on machine vision. Because of the seedling characteristics of leafy vegetables, the unhealthy seedlings cannot be well screened by collecting images from a top-down perspective, so it is necessary to take out a single seedling for accurate identification. As shown in Figures 2A,B, to avoid secondary damage to the seedlings, the screening of healthy seedlings of leafy vegetables is carried out during the transplantation process. After the transplanting manipulator takes out the seedlings, it moves to a position 680 mm opposite the camera, and the camera and the upper plane of the seedling substrate are at the same level. The camera’s position for capturing the image is fixed, and the camera’s position needs to be adjusted before the machine runs. As shown in Figures 2C,D, the image collected by the camera contains six seedlings. The image of a single seedling is cut out through preprocessing, and the six seedlings are numbered from left to right. Then, the seedling images are input into the trained Screening model. If the seedlings are healthy, the manipulator will not move. If the seedlings are unhealthy, the manipulator with the corresponding number will operate independently, the seedlings are moved, and the number of seedlings in the rows and columns is recorded. In this way, the position of the culled seedlings corresponding to the cultivation trough can be calculated to provide coordinate points for subsequent seedling replenishment operations.

### The training of lettuce seedling screening model

#### Image acquisition

In this study, romaine lettuce seedlings were used as the test object, and a 72-hole (6 × 12) Polyvinyl Chloride (PVC) plug tray was used, with an external size of 280 mm × 540 mm. The suitable age for transplantation of lettuce seedlings was 16 days after sowing (Li et al., 2019). Intel RealSense D415 depth camera (Intel Corporation, United States) was used to collect the lettuce seedling image. The image size was 640 × 480. The camera faced the lettuce seedling, and the distance between them was 680 mm. The laptop with Intel Core i5-4210U CPU1.70GHz and 64-bit operating system was connected to the camera. Light-Emitting Diode (LED) strip Light source with the power of 24 W, color temperature of 6,500 K, size of 500 × 50 mm, and model of KM-BRD49242-W was used for lighting. A total of 1,500 lettuce seedling images were collected.

**Figure 3:**
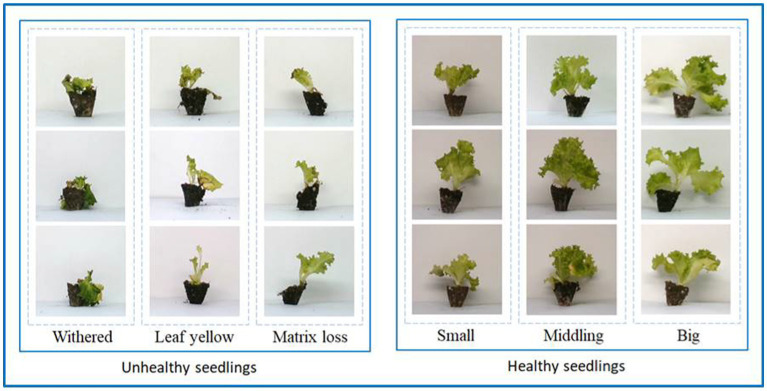
Partial image of the dataset. The healthy and unhealthy seedlings in the dataset are shown.

As shown in Figure 3, according to DB13T 2407–2016 technical specification for lettuce substrate culture, the seedlings with wilting, abnormal growth, substrate loss, and failure to reach the three-leaf one center are classified as unhealthy seedlings, and the rest are healthy seedlings. The unhealthy seedlings are selected and put in an N2 folder, and the healthy seedlings are put in the Y folder. Finally, 1,085 healthy and 415 unhealthy images are obtained. Considering the failure of seedlings picking, it should be a blank image at this time. Therefore, 1,150 blank images are established as a part of the data set and placed in the N1 folder. Due to the small number of samples of unhealthy seedlings, in order to ensure the accuracy of model training, the image of unhealthy seedlings was sheared (25), horizontally flipped, randomly rotated (20), horizontally shifted (0.3), and vertically shifted (0.3). A total of 1,153 pictures of unhealthy seedlings were obtained. A total of 3,388 images were obtained.

#### Data preprocessing

The subsequent operations were carried out on the equipment of AMD Ryzen 53,600× 6-Core Processor 3.80 GHz and 64-bit win10 operating system, equipped with GeForce GTX 1650 graphics card. The software uses Python3.6, Python3.9, and PyCharm.

From the collected images, it can be observed that the backgrounds of the images are slightly different. The different backgrounds may be because the light source is unstable or is affected by ambient light. Therefore, removing the background to extract the seedlings can reduce the computational complexity of deep learning and improve the model detection speed.

This study uses the color extraction method. HSV is a relatively intuitive color model. The parameters of the color in this model are Hue (H), Saturation (S), and Value (V). The corresponding HSV value can be calculated by bringing the RGB value into Equations (1)–(9), thus obtaining the HSV color space table, as shown in Table 1.


(1) $$R^{\prime }=R/255$$

(2) $$G^{\prime }=G/255$$

(3) $$B^{\prime }=B/255$$

(4) $$C_{\max}=\max\left(R^{\prime },G^{\prime },B^{\prime }\right)$$

(5) $$C_{\min}=\min\left(R^{\prime },G^{\prime },B^{\prime }\right)$$

(6) $$\Delta =C_{\max}-C_{\min}$$

(7) $$H=\left\{\begin{array}{c} 0^{{}^{\circ}},\Delta =0\;\\ 60^{{}^{\circ}}\times \left(\frac{G^{\prime }-B^{\prime }}{\Delta }+0\right),C_{\max}=R^{\prime }\\ 60^{{}^{\circ}}\times \left(\frac{B^{\prime }-R^{\prime }}{\Delta }+2\right),C_{\max}=G^{\prime }\\ 60^{{}^{\circ}}\times \left(\frac{R^{\prime }-G^{\prime }}{\Delta }+4\right),C_{\max}=B^{\prime } \end{array}\right.$$

(8) $$S=\left\{\begin{array}{c} 0\;\;C_{\max}=0\;\\ \frac{\Delta }{C_{\max}}\;C_{\max}\neq 0 \end{array}\right.$$

(9) $$V=C_{\max}$$

**Table 1:** HSV color space table.

-	Black	Ash	White	Red		Orange	Yellow	Green	Young	Blue	Purple
H_min_	0	0	0	0	156	11	26	35	78	100	125
H_max_	180	180	180	10	180	25	34	77	99	124	155
S_min_	0	0	0	43		43	43	43	43	43	43
S_max_	255	43	30	255		255	255	255	255	255	255
V_min_	0	46	221	46		46	46	46	46	46	46
V_max_	46	220	255	255		255	255	255	255	255	255

H, S, and V value range corresponds to the color.

**Figure 4:**
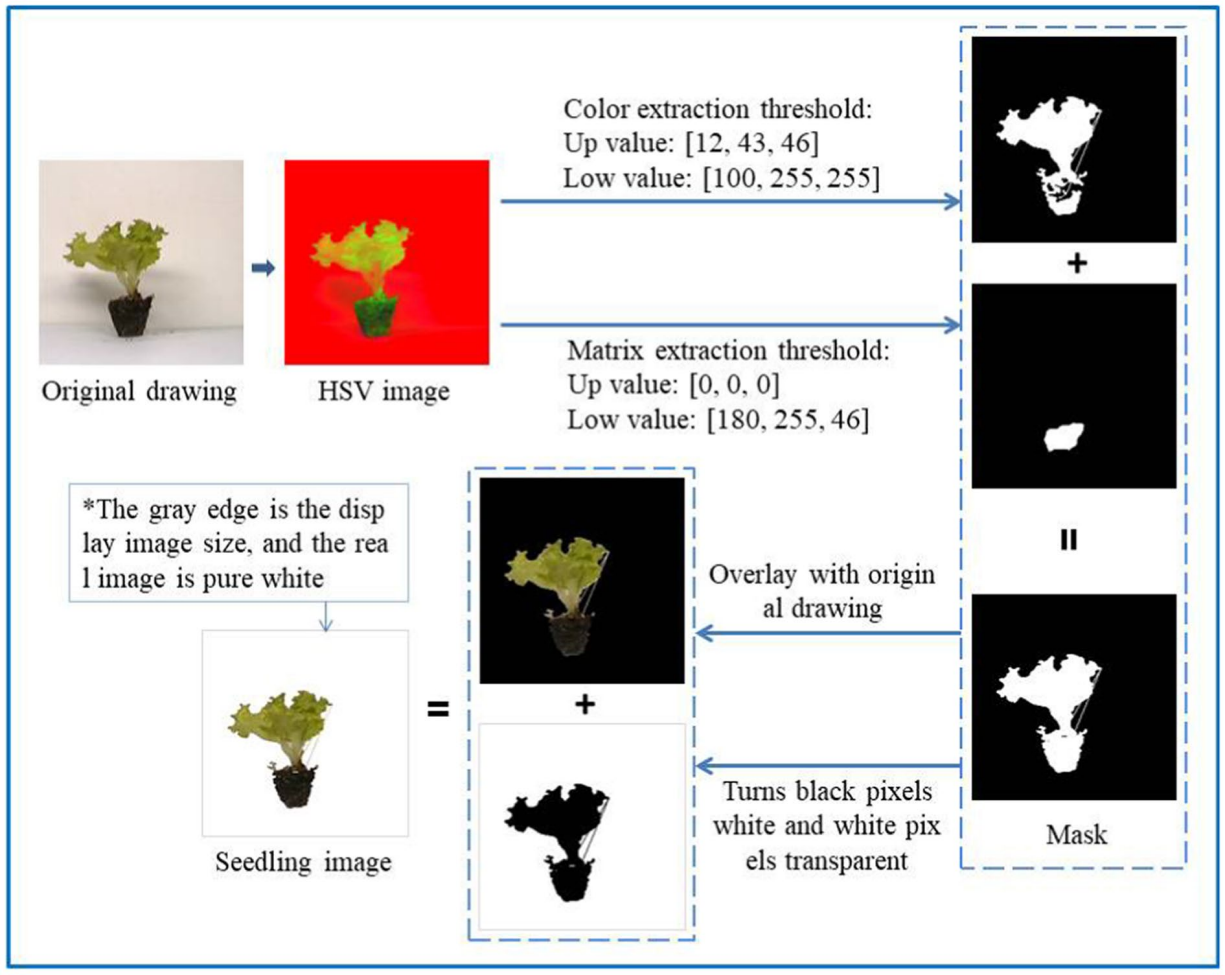
Schematic diagram of extracting seedling image.

As shown in Figure 4, the original image is first converted into an HSV image. Look up Table 1 to determine the threshold for which color must be extracted. Because the background is mainly white and gray, in order to retain as much seedling information as possible, the range of orange, yellow, green, cyan, and blue is selected as the color extraction threshold, and the black range is selected separately as the substrate extraction threshold. The mask image is obtained by summing the two binary images, and the mask image is summed with the original image to obtain an image with a black background. Convert the black pixels in the mask to white pixels, convert the white pixels to transparent pixels, and then sum the image with the black background to get the seedling image.

Since the neural network can only accept an image of a fixed size, all images need to be resized according to the specific neural network requirements. The input image size that the ResNet 18 network can accept is 224 × 224. The path and name of the image are converted into table information and stored in a .csv file. The three categories of images are randomly divided into a training set (60%), validation set (20%), and test set (20%). The images of the training set, validation set, and test set are independent of each other, which can ensure the reliability of the data of the validation set.

#### ResNet 18 network and transfer learning

The process of traditional machine learning feature extraction is complex, but deep learning does not need to extract features manually. Almost all neural networks are open source, and researchers only need to select the appropriate network structure and tune the parameters to make the algorithm reach the optimal state. This study mainly aims to classify, and ResNet 18 is chosen as the base network. There are residual jump connections between layers in the ResNet 18 network, which can introduce forward information, reduce gradient disappearance, and alleviate model degradation. There are 1,000 categories in the ResNet 18 fully connected layer, but the main focus is to identify whether the lettuce seedlings are healthy and whether there are lettuce seedlings. Therefore, according to the classification purpose of this study, a three-classification model is designed. Because there are few classification categories, the initial value of epochs is set to 20 in the following.

**Figure 5:**
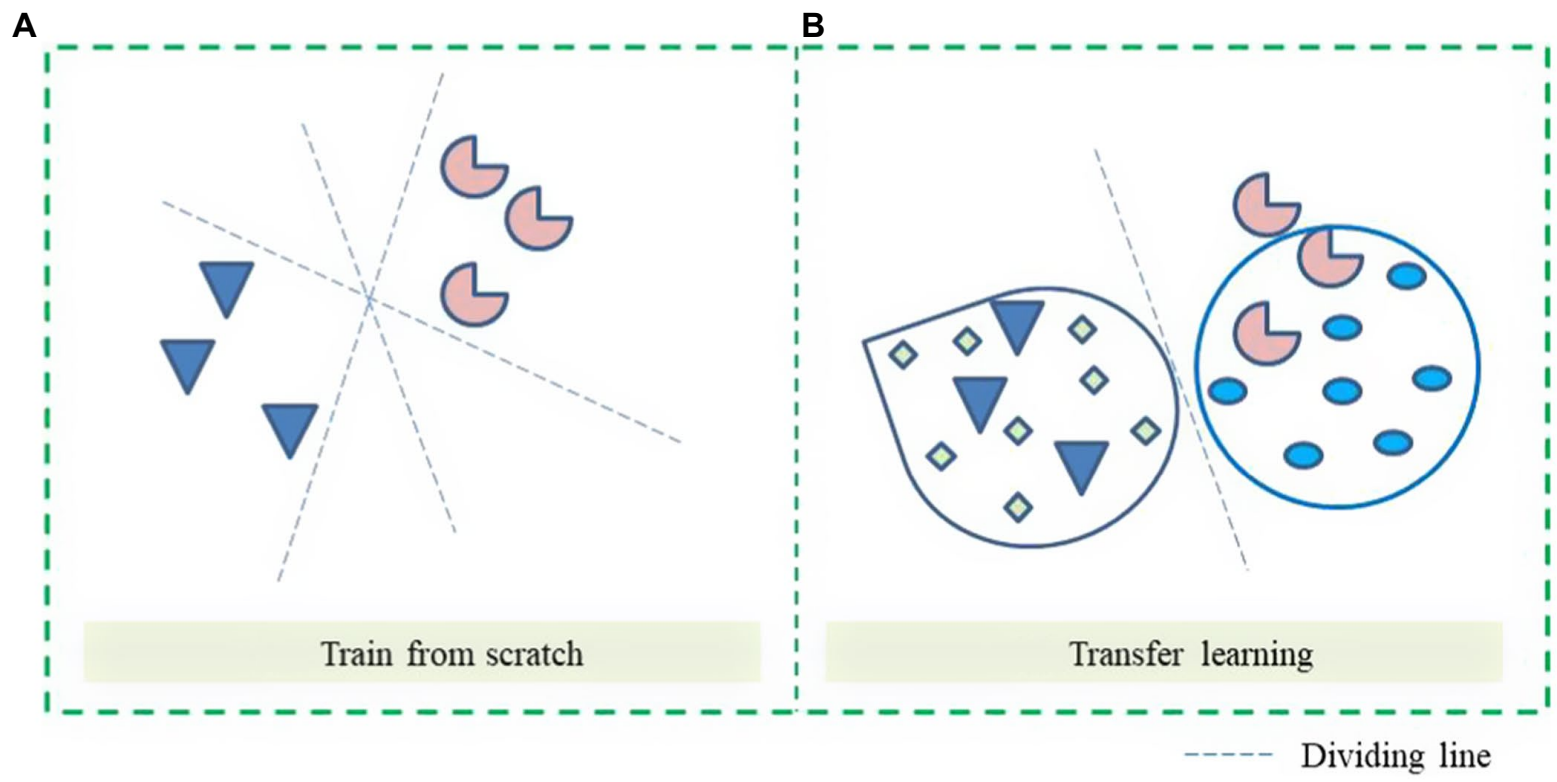
Classification boundaries of different modes. **(A)** Train from scratch. **(B)** Transfer learning.

When the dataset image is relatively small, training from 0 may result in overfitting. As shown in Figure 5A, training from 0, there are multiple possibilities for the dividing line, which may lead to lower validation accuracy. As shown in Figure 5B, transfer learning is selected for training and fine-tuning on the original ResNet 18 network classifier, which is equivalent to starting training on the shoulders of giants, and finally, getting the best classification model.

**Figure 6:**
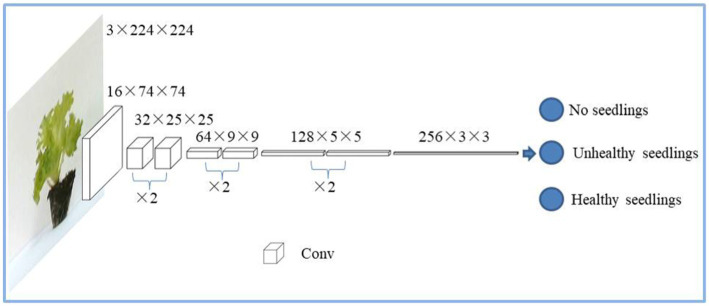
The technical route of identification of lettuce seedlings by the ResNet 18 network.

The custom ResNet 18 network structure is shown in Table 2, with a total of 1,230,275 parameters, and the image segmentation process is shown in Figure 6. The network using transfer learning has 11,178,051 parameters. The model adopts a cross-entropy loss function and Adam optimization algorithm. The initial value of the learning rate is set to 0.001. Set the mini-batch value to 32 and the max training points to 20. Evaluation metrics for qualitative models include accuracy and loss.

**Table 2:** ResNet 18 network structure.

Layer name	Output size	18-layer
Conv1	74 × 74	3 × 3,16
Conv2_x	25 × 25	$\left[\begin{array}{c} 3\times 3,32\\ 3\times 3,32 \end{array}\right]$ ×2
Conv3_x	9 × 9	$\left[\begin{array}{c} 3\times 3,64\\ 3\times 3,64 \end{array}\right]$ ×2
Conv4_x	5 × 5	$\left[\begin{array}{c} 3\times 3,128\\ 3\times 3,128 \end{array}\right]$ ×2
Conv5_x	3 × 3	$\left[\begin{array}{c} 3\times 3,256\\ 3\times 3,256 \end{array}\right]$ ×2
Classification	3	

### Screening of healthy seedlings according to the physical characteristics of seedlings

The use of image processing methods for seedling screening is introduced in this section in order to compare with deep learning methods.

According to DB13T 2,407–2016 lettuce substrate cultivation technical specification standards and the classification method of seedling bases, the screening criteria were designed, and the screening indicators were determined as leaf area and substrate area. First, calculate the leaf area and substrate area of healthy seedlings and unhealthy seedlings obtained in Section “The training of lettuce seedling screening model”, and analyze the minimum leaf area and substrate area of healthy seedlings and the maximum leaf area and substrate area of unhealthy seedlings. If there is an overlap in the data, the seedlings classification is determined according to the standard of DB13T 2,407–2016 Technical Specifications for Substrate Cultivation of Lettuce. The numerical range of each index is shown in Table 3.

**Table 3:** Numerical range of lettuce seedling screening index.

Filter indicators	Healthy seedlings (pixels)	Unhealthy seedlings (pixels)
Leaf area	≥2,300	<2,300
substrate area	≥1,300	<1,300

**Figure 7:**
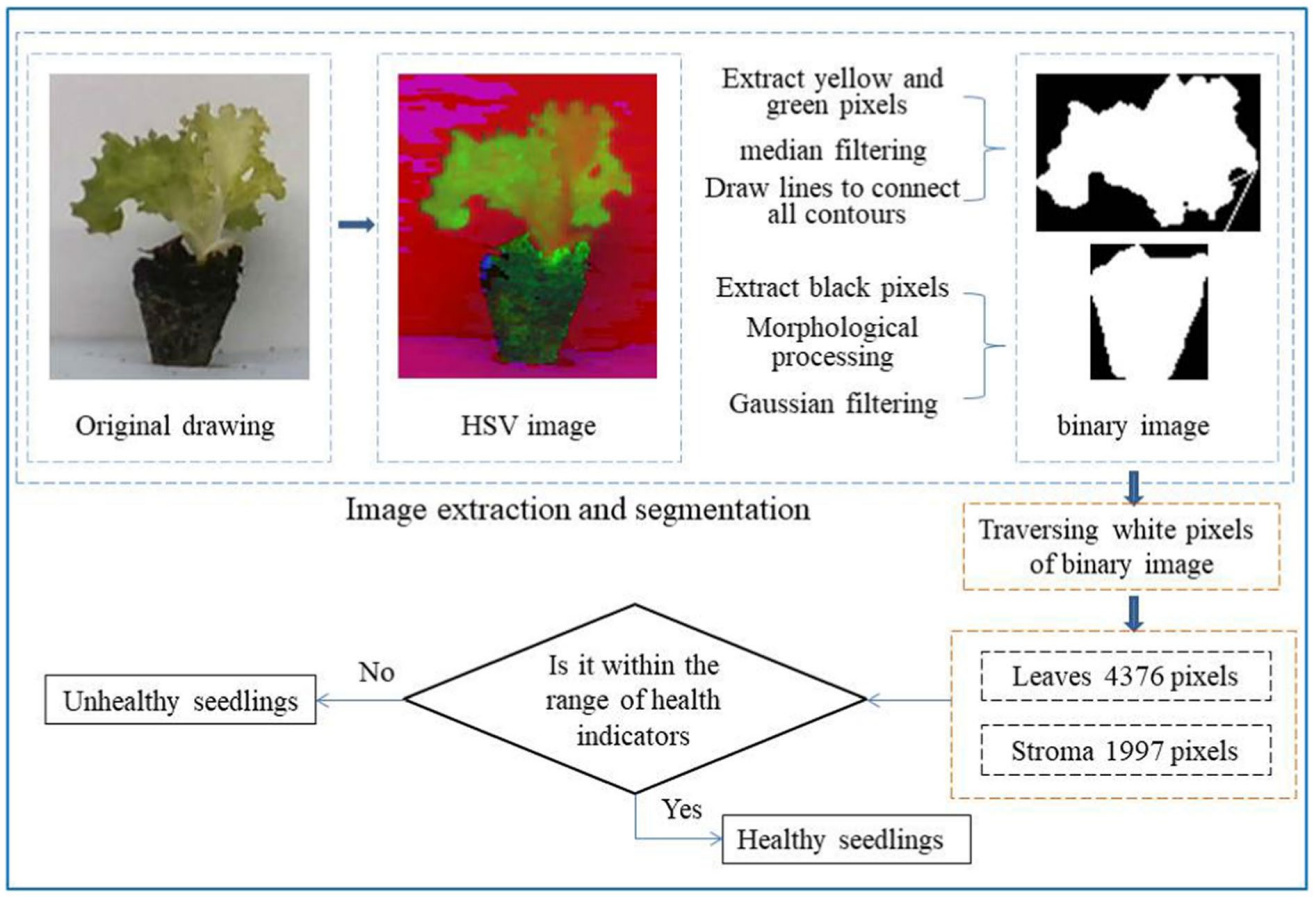
Seedling identification flowchart. The original image is converted to an HSV image, the leaf contour and substrate contour of the seedling are extracted, then the area of the leaf and substrate is calculated, and then whether the area parameter is within the index range can be judged. Healthy seedlings are within the index range, and unhealthy seedlings are not within the index range.

As shown in Figure 7, both leaves and substrate are extracted by HSV color space, and the effect of extracting yellow-green pixels from leaves is better, and only noise points need to be removed. The extraction effect of black pixels in the substrate is poor. After color extraction, morphological expansion and corrosion processing are performed, and then the noise is removed by Gaussian filtering so that the obtained binary image has a high degree of restoration. Then, the white pixels in the binary image are traversed to get the pixel area of the leaf and substrate. Whether it is a healthy seedling is judged according to the index value.

## Results and discussion

### Training model

**Figure 8:**
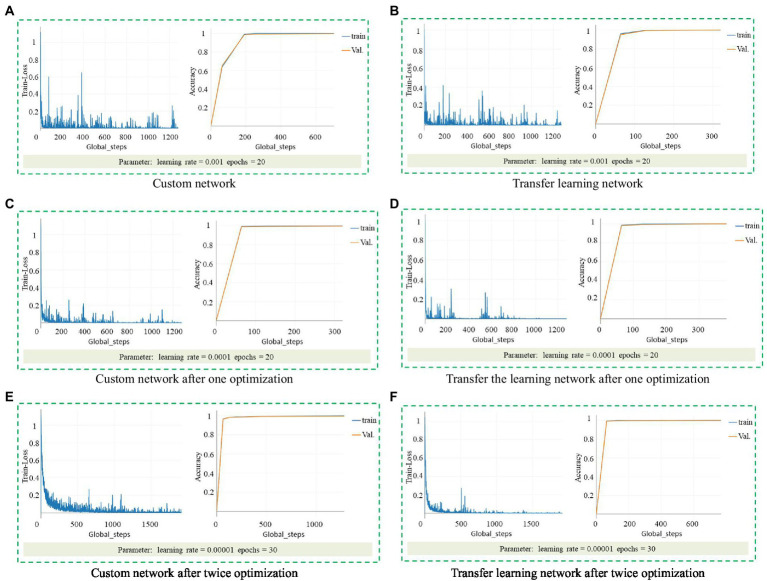
Accuracy and loss results of model training with different parameters. **(A)** Custom network. **(B)** Transfer learning network. **(C)** Custom network after one optimization. **(D)** Transfer the learning network after one optimization. **(E)** Custom network after twice optimization. **(F)** Transfer learning network after twice optimization.

It can be seen from Figure 3 that the appearance of healthy seedlings and unhealthy seedlings is different. Although they can be distinguished manually, the workload is large and does not meet mechanization requirements. This study used the torch as the backend to obtain the ResNet 18 model. A custom ResNet 18 network and a transfer learning strategy were used, and a ResNet 18-based healthy seedling detection model for lettuce seedlings was established. Figure 8 shows the accuracy and loss results of model training with different parameters.

When the learning rate is 0.001, the loss and accuracy results of the custom network are shown in Figure 8A, the highest accuracy of the training set is 99.56%, and the accuracy of the test set is 99.56%. The loss and accuracy results of transfer learning are shown in Figure 8B, the highest accuracy of the training set is 100%, and the accuracy of the test set is 99.71%. In the beginning, the loss value of both dropped sharply, and the accuracy improved significantly, which is a reasonable phenomenon. However, the loss value of the model does not converge, is unstable, and has many glitches, which indicates that the model may not achieve such high accuracy in practical applications.

After setting the learning rate to 0.0001, train again and get the loss and accuracy results of the custom network, as shown in Figure 8C. The highest accuracy of the training set is 99.56%, and the accuracy of the test set is 99.7%. The loss and accuracy results are shown in Figure 8D, the highest accuracy of the training set is 100%, and the accuracy of the test set is 100%. Before the comparison and tuning, it can be seen from the image that the glitches are significantly reduced. The loss image of the transfer learning is stable at 400 steps, but there are still glitches. Due to the limitation of the number of training, it cannot be guaranteed to remain stable after 1,200 steps. The loss image of the custom network still has more glitches.

Set the learning rate to 0.00001, the maximum training point to 30, and then train to get the loss and accuracy results of the custom network, as shown in Figure 8E. The highest accuracy of the training set is 99.41%, and the accuracy of the test set is 99.71%; the loss and accuracy results of transfer learning are shown in Figure 8F, the highest accuracy of the training set is 100%, and the accuracy of the test set is 100%. The loss curve of the custom network shows a downward trend as a whole, but there are still apparent glitches, and the 1000th step of the transfer learning stabilizes the beginning, which is better than the traditional convolutional neural network.

In summary, when the learning rate was 0.00001 and epochs = 30, the model trained by transfer learning was the best detection model.

### Verification of authenticity detection model

Finally, the model is trained by transfer learning when the learning rate is 0.00001 and epochs = 30 are selected for the application. In order to verify the best detection model, 900 lettuce seedlings were newly selected, and the side images of each plant were collected. The best detection model was used to identify these images, and the recognition accuracy was 97.44%. The processing time for a single image is 0.0129 s, and the total time for 900 images is 11.59 s. Table 4 shows the confusion matrix for the authenticity detection model. Table 5 records the FP (False positive), TP (True Position), FN (False negative), and TN (True Negative) values for each class. FP is the model’s negative sample predicted as positive; TP is the positive sample predicted as positive by the model; FN is the positive sample predicted as negative by the model; and TN is the negative sample predicted as negative by the model.

**Table 4:** Confusion matrix of the authenticity detection model.

Confusion matrix		True		
		Healthy seedlings	Unhealthy seedlings	No seedlings
Forecast	Healthy seedlings	439	11	0
	Unhealthy seedlings	12	138	0
	No seedlings	0	0	300

**Table 5:** FP, TP, FN, and TN values for each class.

–	FP	TP	FN	TN
Healthy seedlings	11	439	12	438
Unhealthy seedlings	12	138	11	739
No seedlings	0	300	0	600

Calculate each category’s precision rate, recall rate, and F1 value through Equations (10)–(12). The results are shown in Table 6. The closer the results of the precision rate, recall rate, and F1 value are to 100%, the better the effect of the model is. It can be seen from Table 6 that all indicators of healthy seedlings are above 97%, those of unhealthy seedlings are above 92%, and the indicators of no seedlings are 100%. In general, the effect of this model is good.


(10) Precision=TP/(TP+FP)

(11) Recall=TP/(TP+FN)

**Table 6:** Results of various precision rates, recall rates, and F1 values.

–	Healthy seedlings	Unhealthy seedlings	No seedlings
Precision	97.56%	92%	100%
Recall	97.34%	92.62%	100%
F1	97.4%	92.31%	100%

The comprehensive index for balancing precision rate and recall rate:


(12) $$F1=\frac{2*\mathrm{Precision}*\mathrm{Recall}}{\mathrm{Precision}+\mathrm{Recall}}$$

**Figure 9:**
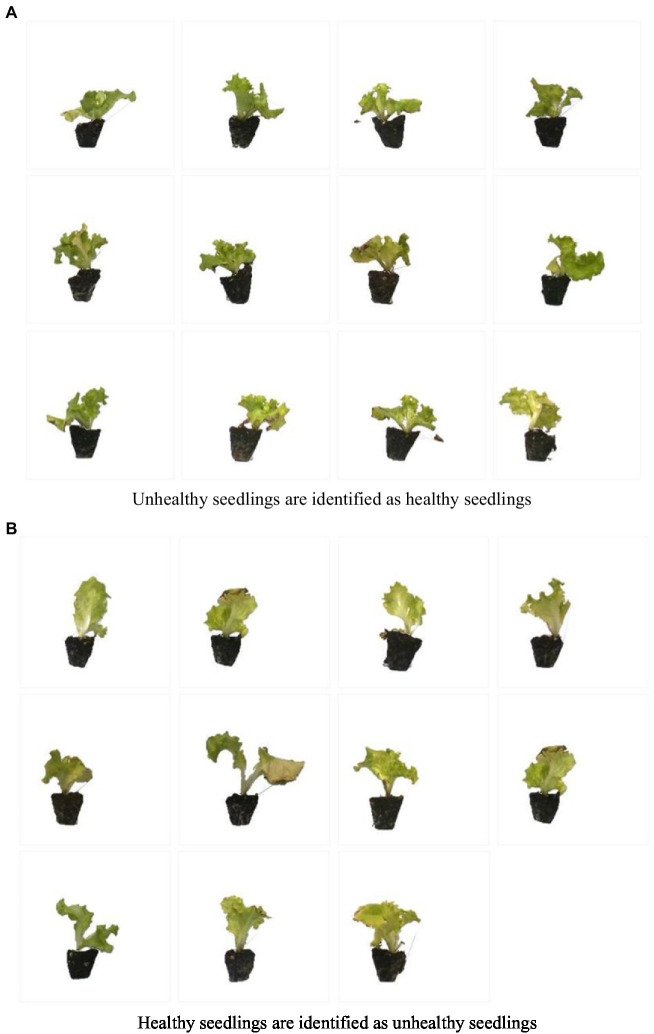
The image was identified as wrong. **(A)** Unhealthy seedlings are identified as healthy seedlings. **(B)** Healthy seedlings are identified as unhealthy seedlings.

Figure 9 shows the visualization of validation results. Twenty-three seedlings were identified incorrectly. It is speculated that unhealthy seedlings were identified as healthy seedlings because the leaf area or erectness met the requirements but did not meet the standard of three leaves and one heart. Healthy seedlings are identified as unhealthy because the leaf area or height does not meet the requirements but meets the standard of three leaves and one heart. From the images in Figures 9A,B, it can be seen that the size of these seedlings is not much different, the difference lies in whether they are three leaves and one heart, and the seedlings that do not reach the three leaves and one heart are unhealthy seedlings. These seedlings are on the line between healthy and unhealthy seedlings, causing the model not to recognize them well.

### Authenticity detection model of lettuce seedlings based on physical characteristics of lettuce seedlings

Lettuce seedlings’ leaf area and substrate area were extracted and compared with the index values. If both leaf area and substrate area met the criteria of healthy seedlings, it was judged as healthy seedlings. If the values were all 0, there were no lettuce seedlings. In other cases, it was judged as an unhealthy seedling. A total of 900 seedling images were analyzed, and the accuracy rate was 89.33%. The processing time for a single image is 0.0427 s, and the total time for 900 images is 38.46 s. Table 7 shows the confusion matrix of the physical feature authenticity detection model. Table 8 records each class’s FP, TP, FN, and TN values.

**Table 7:** Confusion matrix of physical property authenticity detection model.

Confusion Matrix		True		
		Healthy seedlings	Unhealthy seedlings	No seedlings
Forecast	Healthy seedlings	399	41	0
	Unhealthy seedlings	55	105	0
	No seedlings	0	0	300

**Table 8:** FP, TP, FN, and TN values for each class.

–	FP	TP	FN	TN
Healthy seedlings	41	399	55	405
Unhealthy seedlings	55	105	41	699
No seedlings	0	300	0	600

Calculate each category’s precision rate, recall rate, and F1 value through Equations (10)–(12), and the results are shown in Table 9. The closer the results of the precision rate, recall rate, and F1 value are to 100%, the better the effect of the model is. It can be seen from Table 6 that the indicators of healthy seedlings are about 89%, those of unhealthy seedlings are about 68%, and the indicators of no seedlings are 100%. In general, the effect of this model is not very good.

**Table 9:** Results of various precision rates, recall rates, and F1 values.

–	Healthy seedlings	Unhealthy seedlings	No seedlings
Precision	90.68%	65.63%	100%
Recall	87.89%	71.92%	100%
F1	89.26%	68.63%	100%

**Figure 10:**
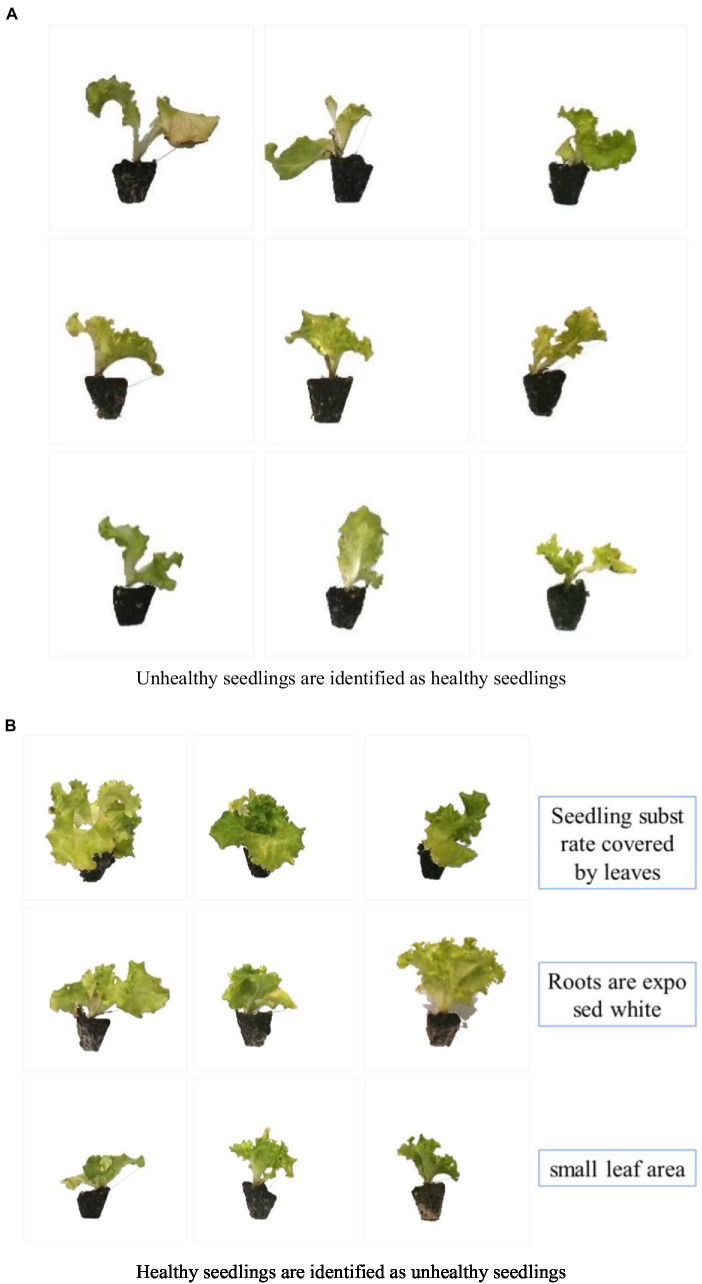
The image was identified as wrong. **(A)** Unhealthy seedlings are identified as healthy seedlings. **(B)** Healthy seedlings are identified as unhealthy seedlings.

As shown in Figure 10, the identified error diagram is displayed. A total of 96 images were identified incorrectly. Unhealthy seedlings were identified as healthy because the leaf area met the requirements, but the seedlings did not meet the standard of three leaves and one heart. There are three reasons why healthy seedlings are identified as unhealthy seedlings: (1) The leaves block the seedling substrate, so the correct substrate area cannot be calculated; (2) because of the vigorous root system and the root agglomeration mechanism, the root system outside the substrate is prominent, resulting in the extracted substrate area being less than the actual area; and (3) the leaf area is small, but the seedlings meet the standard of three leaves and one heart. The seedlings on the dividing line between healthy and unhealthy seedlings are not easy to be correctly identified by the model.

## Discussion

From the F1 indicators in Tables 6, 9, the screening of models trained by deep learning had a good effect. Deep learning did not require the users to perform complex feature extraction steps. Appropriate network structures and optimization algorithms were selected according to needs, and parameters were optimized. Finally, the successful training model can be applied. It should be noted that there was a slight gap in the model’s accuracy for each training, and finally, the highest accuracy was selected for the application. The effect of screening based on the physical characteristics of lettuce seedlings is not good, mainly because of the screening with fixed characteristic parameters. In some exceptional cases, such as the leaves blocking the substrate and the root system leak, more are easy to judge wrong and are not flexible.

The seedlings on the dividing line between healthy and unhealthy seedlings are not easy to be correctly identified by the model. The main reason is that the leaf area of these seedlings meets the standard but does not meet the standard of three leaves and one heart, and it is easily misidentified. When establishing a deep learning data set, there is the influence of subjective consciousness, which leads to the blurred classification of seedlings, which is also one of the reasons for the wrong identification of seedlings. In the follow-up research, in order to improve the generality and accuracy of the authenticity detection model, it is necessary to add as many standard samples from different changing environments as possible to the model to enhance its robustness.

Table 10 records the image processing time of the two models. The model trained with the original image is the model trained with the image in Figure 3. The model trained by extracting seedlings alone refers to the model trained by obtaining the background-removed seedling images through the preprocessing in Section “Data preprocessing.” It can be seen that the model trained from the image with the background removed dramatically reduces the processing time because after the seedlings are extracted separately, the background of the seedling image is white. Other information other than the seedlings is removed, reducing the calculation of the model quantity. The image processing time is significant for the automation of seedling transplanting machinery, and the image processing time directly affects the overall transplanting efficiency, so the smaller the image processing time, the better. The seedling transplanting workload is small, and screening during the transplanting process is feasible. When the transplant workload is large, it can be considered to separate the screening work from the transplant work to ensure transplant efficiency. This is the same as the process concentration and process decentralization in machining.

**Table 10:** Time to process images for different models.

Model		Average processing time of the single picture (s)	Total processing time of 900 images (s)
Deep learning model	Model trained with original image	0.0934	84.1
	Model trained by extracting seedlings alone	0.0129	11.59
Physical characteristic model		0.0427	38.46

The method of collecting images from the side of seedlings for training proposed in this study has more vital adaptability than that of collecting images from the top view for training. Because the two factors of plug tray specification and leaf vegetable seedling type did not affect the feasibility of this method, this method can also be applied to other specifications of plug tray cultivated seedlings. The screening of leaf vegetable seedlings can be realized. It only needs to replace the data set and retrain to get a new model for the application. If images are collected from a top-down perspective for training, the size of the plug tray is required to be large so that a single seedling image can be extracted entirely.

## Conclusion

1. In this study, deep learning and transfer learning strategies were adopted, and the Resnet 18 network was used to establish the identification model of healthy lettuce seedlings. In discrimination, the detection accuracy of the optimal model was more than 100%, and the model loss remained about 0.005, which was better than the recognition model based on physical characteristics.
2. Another 900 seedling samples were tested, and the recognition accuracy was as high as 97.44%. The model was efficient and straightforward. The average processing time for a single image was 0.0129 s, and the 900 samples consumed 11.59 s. The background removal technology is used to extract seedling images for model training, which significantly reduces the amount of model computation, reduces the processing time of a single image, and further improves the efficiency of seedling transplanting machinery.
3. This method can solve the problematic screening of dense plug seedlings by selective transplanting machinery.

This method is to carry out seedling screening during the transplantation process, and the screening accuracy is 97.44%. This method can be applied to the scene of graded transplantation, which is convenient for accurate management of fertilization and other operations in the later stage of seedlings. It can also be applied to grading finished vegetables or flowers, which is convenient for graded sales. It will be the content of further research to establish corresponding data sets for different application scenarios for training and select the optimal model for the application.

## Data availability statement

The raw data supporting the conclusions of this article will be made available by the authors, without undue reservation.

## Author contributions

XJ and LT contributed to the conception and method of the study. RL and JJ organized the database. JL performed the statistical analysis. XJ wrote the first draft of the manuscript. LT, RL, JJ, and JL wrote sections of the manuscript. All authors contributed to the article and approved the submitted version.

## Funding

This work was supported by the National Key Research and Development Program of China Project (No. 2021YFD2000700), the National Natural Science Foundation of China (No. 51875175), the Natural Science Foundation of Henan Province (No. 202300410124), and the Special projects for Industrial Foundation Reconstruction and High-quality Development of Manufacturing Industry in MIIT.

## Conflict of interest

The authors declare that the research was conducted in the absence of any commercial or financial relationships that could be construed as a potential conflict of interest.

## Publisher’s note

All claims expressed in this article are solely those of the authors and do not necessarily represent those of their affiliated organizations, or those of the publisher, the editors and the reviewers. Any product that may be evaluated in this article, or claim that may be made by its manufacturer, is not guaranteed or endorsed by the publisher.
